# The Lifestyle of Saudi Medical Students

**DOI:** 10.3390/ijerph18157869

**Published:** 2021-07-25

**Authors:** Khalid A. Bin Abdulrahman, Ahmad M. Khalaf, Fahad B. Bin Abbas, Omran T. Alanezi

**Affiliations:** College of Medicine, Imam Mohammad Ibn Saud Islamic University (IMSIU), Riyadh 13317-4233, Saudi Arabia; amrkhalaf@sm.imamu.edu.sa (A.M.K.); fbsbin@sm.imamu.edu.sa (F.B.B.A.); otalanazy@sm.imamu.edu.sa (O.T.A.)

**Keywords:** Saudi Arabia, lifestyles, medical students, medical education

## Abstract

This study was conducted to investigate medical students’ lifestyle habits, including sleep quality, eating and drinking patterns, physical activity, and social status. *Method*: This research project is part two of a multi-institutional cross-sectional observational study conducted among medical students from six medical colleges in Saudi Arabia between September and December 2019. *Results*: 675 medical students were enrolled electively into the lifestyle study. About half of this number were male students and the majority were aged 18–24 years. Most students (87.6%) slept between 4–8 h a day and over 44% were dissatisfied with their sleep. Only 28.1% had three meals a day; about 40% of them usually or always skipped breakfast. A total of 44% usually or always ate fast food and 44.7% drank 2 L of water per day. Moreover, male students were significantly consuming more fast food than females, *p* < 0.001. The majority (63.3%) revealed they usually or always drink black coffee daily. Females were significantly more inclined to regular coffee consumption than males, *p* < 0.001. Only 4.3% exercised for 30 min or more daily. The majority (65%) of the students were introverted; they had few close friends. Yet, 81% were somewhat satisfied or satisfied with their social life. Male students were significantly more satisfied with their social life than females, *p* = 0.001. Only 4.6% smoked cigarettes daily whereas 7.1% smoked e-cigarettes daily. In contrast, only 0.3% used shisha (hookah) daily. Male medical students were substantially more inclined to e-cigarette use than females (*p* < 0.001). The top five leisure activities of a medical student were surfing social media (75.9%), watching movies (61.3%), hanging out with friends (58.1%), spending time with their family (55.4%), and browsing the Internet (53.6%). Female medical students were significantly more inclined to surf social media than male medical students, *p* = 0.022; also, watching movies was preferred for females compared to males, *p* = 0.006. *Conclusion*: This study revealed that the majority of these medical students in Saudi Arabia exhibited healthy lifestyles to some extent, and these health-promoting behaviors differed based on sex, especially concerning physical activity and eating patterns. The findings of this study provide relevant information for future actions that will be geared towards effectively decreasing the occurrence of chronic illnesses and improving future doctors’ well-being.

## 1. Introduction

The term lifestyle describes a particular person or group of people and the living conditions, behaviors, and habits that are typical of them or are chosen by them [1]. Lifestyle habits are important indicators of future well-being, productivity, and life expectancy. In a recently published meta-analysis of prospective cohort studies, younger adults have more cardiovascular benefits from combined healthy lifestyle factors [2]. Since doctors represent a unique and remarkable human resource within a nation, improving and safeguarding physicians’ health through lifestyle modification is a health preservation strategy that is beneficial to society. Conservation of doctors’ health and well-being should begin when they start their training in medical school.

Most healthy lifestyle habits are cultivated early, are challenging to change, and are retained throughout life; the earlier such an action begins, the more compelling it will be [3]. It is usually a common perception that medical students have better information about healthy lifestyle habits when compared to non-medical students. However, there is no proof whatsoever to show that this information translates into preserving excellent well-being practices [4,5]. Lifestyle habits among medical students could be affected by worries, just as stress can affect students’ academic accomplishments. Propensities in any group of individuals will culminate in a positive outcome: healthy habits, or contrarily, prompting lifestyle disorders [6]. A sedentary lifestyle has precipitated an increase in the prevalence of dyslipidemia, obesity, and cardiovascular diseases [7,8].

Evaluating the quality of life of medical students can allow us to better understand their general well-being and lead to appropriate interventions to promote students’ quality of life. This could prevent psychological distresses and other pitfalls threatening students’ professional life, and ultimately improve the quality of care provided to future patients [9,10,11]. Almost all Saudi medical colleges have developed their curricula. The majority have adopted the new trends in teaching and learning that focus on active learning, problem-based learning, vertical and horizontal integration, and community-based oriented education [12,13,14,15].

There are many publications on lifestyle habits among university students [16,17,18,19,20,21]. However, in Saudi Arabia (KSA), there are scarcely any investigations depicting medical students’ knowledge and their view of healthy lifestyle habits [22]. Consequently, this study aims to describe medical students’ lifestyle habits in six medical schools in KSA. 

## 2. Materials and Methods

### 2.1. Study Design

This research project is part two [23] of a multi-institutional survey. The study has two sections, a cross-sectional observational followed by research analysis on sex differences. The study was conducted among medical students at various medical colleges in Saudi Arabia. The study was conducted according to the guidelines of the Declaration of Helsinki and approved by the Institutional Review Board of the Imam Mohammad Ibn Saud Islamic University IRB committee number 68-2019 dated 17 November 2019.

### 2.2. Participants and Sampling

All medical students of either sex of the pre-clinical and clinical years were invited by email through the vice-dean of academic affairs of the six medical colleges in Saudi Arabia. Two reminder emails and SMS weblink messages were sent to enhance the response rate. The data were collected from the medical students who responded from the six medical colleges; the four colleges situated in the Riyadh region include Imam Mohammad Ibn Saud Islamic University (Public University), King Saud Bin Abdulaziz University for Health Sciences (Public University), Alfaisal University (Private University), and King Saud University (Public University). King Abdulaziz University (Public University) is in Jeddah and Qassim University (Public University) in the Qassim region. 

### 2.3. Study Questionnaire

The questionnaire was designed to study medical students’ lifestyle habits, including sleep quality, eating and drinking routine, physical activity, social status, and leisure activities. 

The students were informed about the purpose of the study. Instructions regarding the questionnaires were provided to volunteering students. The confidentiality of information was also ensured. Once students voluntarily signed the informed consent, they were requested to fill in the study questionnaire. 

The questionnaire was composed of three parts. Part I addressed the sociodemographic data, including age, sex, marital status, level of education, level of study, residence, grade point average, and university name. 

Part II covered the smoking habits and the response ranged from 0 to 5 points, where 0 indicates never and 5 indicates daily. 

The sleeping duration response ranged from 0 to 4 points, where 0 indicates the duration <4 h and 4 indicates >8 h. The sleeping satisfaction response ranged from 0 to 5 points, where 0 indicates very dissatisfied and 5 indicates very satisfied. Falling asleep easily response ranged from 0 to 5 points, where 0 indicates very difficult and 5 indicates very easily.

Eating habits included daily meals and responses ranged from 0 to 7 points, where 0 indicates breakfast and dinner and 7 indicates lunch only. Fast-food consumption and skipping breakfast response ranged from 0 to 5 points, where 0 indicates never and 5 indicates always. Daily drinking of two liters of water and drinking coffee per day responses ranged from 0 to 5 points, where 0 indicates never and 5 indicates always.

The physical exercise ≥30 min response ranged from 0 to 8 points, where 0 indicates never and 8 indicates daily. The student satisfaction with social life response ranged from 0 to 3 points, where 0 indicates not satisfied and 3 indicates satisfied. The questionnaire also included a question on what leisure activities medical students took part in. 

Part III was devoted to studying habits and time management practices based on an already published piece of work [23]. The questionnaire was subjected to pilot testing by 25 students; some questions were modified accordingly. All 900 randomly selected students from all six medical schools were emailed to participate and reminded by emails and via an SMS web link.

### 2.4. Statistical Data Analysis

The means and standard deviations describe the continuous variables, and frequency and proportions were used to describe the categorical variables. Histograms and the Kolmogorov–Smirnov test were used to assess the normality of continuous variables. The chi-squared test (χ^2^) of independence was used to assess the correlation between categorical variables, and the likelihood ratio adjusted chi-squared test and its associated *p*-values were quoted when the statistical assumptions of these statistical tests were violated. Multiple response dichotomy analysis was used to describe the questions measured with multiple option selection (select all that applies). SPSS IBM V20 (IBM, Armonk, NY, USA) was used for the data analysis, and the statistical significance alpha level was considered at 0.050 level. Excel was used for creating figures and depictions.

## 3. Results

A total of 675 out of 900 medical students responded (75%) and were enrolled in the lifestyle study. They were from six medical schools in different Saudi universities. Table 1 summarizes the sociodemographic and academic characteristics of the study population.

Table 2 shows the sleeping, eating, drinking, and exercising habits of the medical students. Most students (87.6%) slept between 4–8 h a day. Over 44% were dissatisfied with their sleep. However, 18.4% were happy with their sleep. Furthermore, 17.6% found it difficult or very difficult to fall asleep. Only 28.1% had three meals a day. About 40% of them usually or always skipped breakfast, while 44% usually or always ate fast food. Moreover, 44.7% drank 2 L of water per day. 

Table 3 displays the descriptive analysis of the medical students’ social activities and smoking habits. The majority (65%) of the students were introverted, they enjoyed being isolated or they had a few close friends only. A total of 81% were somewhat satisfied or satisfied with their social life. Only 4.6% smoked cigarettes daily, whereas 7.1% smoked e-cigarettes daily. In contrast, only 0.3% used shisha (hookah) daily. 

Table 4 showed the bivariate analysis of lifestyle characteristics across sex. The daily eating habits differed significantly between male and female medical students, *p* < 0.001. Moreover, male students were significantly more inclined to consume fast foods than females, *p* < 0.001. The majority (63.3%) revealed they usually or always drink black coffee daily. Females were significantly more inclined to regular coffee consumption than males, *p* < 0.001. Surprisingly, only 4.3% exercised for 30 min or more daily. Male students were significantly more satisfied with their social life than females, *p* = 0.001. Male medical students were significantly more inclined to daily smoking than female medical students, *p* = 0.001 according to a likelihood ratio adjusted chi-squared test. Additionally, male medical students were substantially more inclined to e-cigarette use than females (*p* < 0.001).

**Figure 1:**
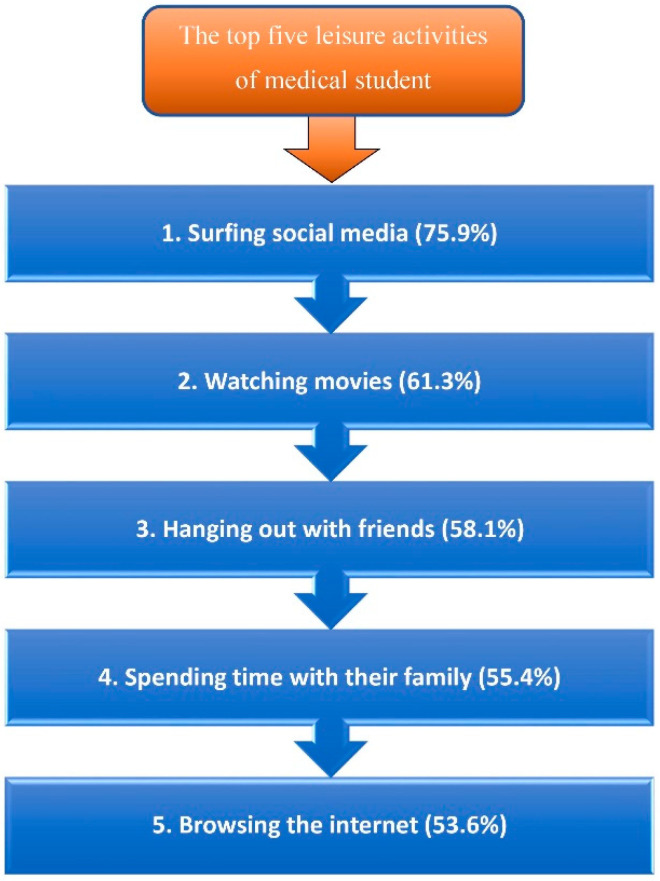
The top five leisure activities of medical student.

The top five leisure activities of the medical students are listed in Figure 1. Female medical students were significantly more inclined to surf social media than male medical students, *p* = 0.022; also, watching movies was preferred for females compared to males, *p* = 0.006 according to the chi-squared test of association. Regarding the medical students’ social, smoking, and leisure activity characteristics, the analysis findings suggested that social type (extroversion) did not differ significantly between male and female students, *p* = 0.0716. There was no significant association between lifestyle habits and other demographic characteristics, namely marital status, residence location, designated school of medicine, and GPA. Part one of the study questionnaire focused on the study habits of highly effective medical students. The results showed a significant correlation between study habits and students’ academic accomplishments. The top ten study habits of highly effective medical students were described in detail in a previous publication [23].

## 4. Discussion

College life is a conceivably important objective for the endorsement of a youthful adult populace’s healthy lifestyle. It is not surprising that this study showed statistically significant differences between male and female students in Saudi medical colleges. Male students tend to go out more, play sports, enjoy spending time with friends, play electronic games, surf the Internet, and use social media. At the same time, female students tend to spend most of their time studying, preparing for exams, isolating themselves for the sake of achievement, surfing the net, intermittent shopping, and having limited friendships. As Islam is the official religion of Saudi Arabia, the culture and religion impact Saudi medical students. Female students had relatively fewer outdoor activities as compared to male students. This could be explained by the fact that Saudi families sympathize with supporting and protecting their female medical students. Therefore, they tend to prove that they can achieve as much as their counterpart male students. However, many factors can explain this difference, such as the stable extrinsic environment in Saudi Arabia in terms of political, economic, religious, and well-balanced cohesive society supporting students’ psychological well-being [9].

The sex-specific distinction was perceived in terms of the students’ nutritional and lifestyle knowledge and attitude, as shown in previous studies, which reported that female students accomplished better scores than male students on nutritional knowledge and perspective [24]. There was not much difference in hours slept per day between the two sexes; 49.2% of the female students slept 4–6 h daily compared to 43.6% of the male students. Furthermore, 37.9% of the females slept 6–8 h per day compared to 44.4% of the males (*p* = 0.258). Regarding sleep satisfaction, only a small proportion was satisfied in the female group (15.3%) and the male group (20.9%) (*p* = 0.103). Most of the students had no problems falling asleep at night (*p* = 0.182). Another similar study conducted on medical students showed that the average sleep duration was 6–8 h [25]. In 2002, the National Sleep Foundation poll revealed that Americans’ average sleep duration was 6.9 h per weeknight [26], which was considered a positive habit. For the nutritional status, 23.6% of female students had breakfast, lunch, and dinner while in the male group, the proportion was 31.8%. However, the number of female students who had lunch and dinner was 19.9% compared to 34.8% for male students. Furthermore, female students had a higher proportion for breakfast and lunch only (27.5%) compared to 11% for the males (*p* ≤ 0.001). Regarding fast food consumption, both groups had similar proportion values since the majority of both sexes chose the options “sometimes” and “usually” (*p* ≤ 0.001). However, this does not pertain to the daily consumption of 2 L of water since 30.9% of females rarely drank 2 L of water compared to 16% of males. Furthermore, 18.6% of the female students usually drink 2 L of water compared to 30.2% of male students (*p* ≤ 0.001). Interestingly, only a small proportion of both groups always skip breakfast (*p* = 0.528). These results are comparable to a study conducted on Chinese medical students. The outcomes showed that most students (83.6%) reported taking meals regularly, with 79.0% eating meals three times daily. There were no differences in terms of sex. However, a noteworthy sex difference was observed in response to questions bordering on how often they had breakfast. A total of 66.8% of males and 82.3% of females reported having breakfast regularly (*p* < 0.0006) [2]. The skipping of breakfast has been associated with lower nutritional status and cardiovascular diseases [27]. It has also been reported that inappropriate breakfast habits may lead to the appearance and progression of obesity [28]. Regarding fast food consumption, a study was conducted on Saudi medical students in Dammam. The results showed that 91.3% admitted to eating fast foods. Approximately 25% ate fast food twice or less every week, while 85% of males ate it three times or more every week [5]. The evidence has clearly shown that fast food consumption is well related to the onset of cardiovascular disease [29,30]. Daily caffeine consumption between the two groups did not differ much; the proportion of the female students who drank coffee daily was 57.1% compared to 39.3% of the male students (*p* ≤ 0.001). The results aligned with other studies which also showed that medical students drank one coffee per day [27,31]. Regarding physical activity, we asked the students if they engaged in exercise for 30 min or more. The results showed that most of the female students rarely engaged in exercise (29.2%) compared to 21.4% among the male students. We also found that only 16.9% of the females exercised a few times per week compared to 21.4% of male students. Surprisingly, the study showed that 18.3% had never exercised among the female group compared to 15.5% among the male group (*p* = 0.138). Diminished degrees of physical activity are associated with an increase in the prevalence of an overweight condition, obesity, and diet-related non-communicable diseases among the young [32,33]. The two main barriers identified by medical students from most investigations were stress and lack of time [19,21]. Different studies have suggested that most medical students report not having sufficient time to exercise and eat healthier meals because of their courses and clinical rotation demands [32,34]. Regarding the social status of the students, female students were primarily introverted (65.8%) and male students were also predominantly introverted (64.4%) (*p* = 0.716). The female students also expressed that they were somewhat satisfied with their social life (45.8%) compared to 42% in the male group; 30.6% of female students were satisfied compared to 43% in the male group (*p* = 0.001). Introverted students may consider specific teaching settings more complicated than extroverted and outgoing students; these settings include group discussions with more than a few colleagues, locations where the establishment of fast relationships with students is expected, and environments where thoughts need to be offered rapidly or decisively [35]. There was not much difference between the two groups vis-à-vis smoking habits since the majority in both groups do not smoke cigarettes (*p* = 0.001), electronic cigarettes (*p* ≤ 0.001), or shisha (*p* = 0.032). A prior study indicated that friends’ influence is the principal reason for smoking among college students, followed by stress, curiosity, and imitation of smokers [36]. In different studies carried out in other countries, peer pressure was also identified as a reason for smoking [37,38]. Thus, peer pressure among students is a definite factor in beginning the habit. Concerning psychological pressure, comparative findings were additionally reported in different nations [39]. Furthermore, most non-smokers reported that religion was the principal factor against smoking, followed by well-being consideration and smoking control policies [36]. During spare time activities, the proportion of female students who browsed social media was somewhat similar (80.1%) compared to male students (72.5%) (*p* = 0.022). The number of male students who played video games was 51.1% compared to 15% of the female students (*p* ≤ 0.001). The proportion of female students who engaged in their favorite hobbies was slightly higher (30.2%) compared to the male students (27.5%) (*p* = 0.442). Furthermore, the number of females who read non-medical books was 33.6% compared to 21.7% of the male group (*p* = 0.001). It has been recognized that the nature of clinical training is generally distressing, and the pressure cannot be reduced [40]. Thus, it is imperative to analyze medicinal and preventive stress intervention practices in the medical curriculum. One investigation suggested that students with negative perspectives toward free-time activities were the ones who reported encountering the most pressure. Since medical training stresses do not significantly reduce in actual practice, students who fail to figure out how to adapt to stress early may become miserable, unhealthy physicians [41]. 

## 5. Limitations

No validated instruments were used, and questions arose from a brainstorming session created by authors. All data were self-reported and subject to reporting bias. Furthermore, the investigation report provided here was established based on the bivariate comparison (female versus male medical students). Another limitation is that a student may comprehend each question distinctly. Thus, their answer might be based on their understanding of the question.

## 6. Conclusions

The findings revealed that the vast majority of the medical students in Saudi Arabia exhibit healthy lifestyles to some extent, and these health-promoting behaviors differed by sex, especially regarding physical activity and eating patterns. The outcomes obtained here provide relevant information for future actions geared towards effectively decreasing chronic illnesses and improving population well-being.

## Figures and Tables

**Table 1:** The medical students’ sociodemographic and academic characteristics. *N* = 675.

Variables		Frequency	Proportion
Sex	Female	301	44.6
	Male	374	55.4
Age	18–24	614	91
	25–34	61	9
Marital status	Single	654	96.9
	Married	61	3.1
Place of residency	Student dormitory	24	3.6
	Family residency	619	91.7
	Privately rented house	32	4.7
College of medicine	IMSIU	131	19.4
	Alfaisal University	143	21.2
	King Abdulaziz University	110	16.3
	KSAU-HS	108	16
	King Saud University	69	10.2
	Qassim University	114	16.9
Level of education	1st year	150	22.2
	2nd year	121	17.9
	3rd year	118	17.5
	4th year	90	13.3
	5th year	136	20.1
	Internship year	60	8.9
Level of study	Pre-clinical phase (years 1–3)	271	40.1
	Clinical phase (years 4–6)	404	59.9
Academic GPA	<4 out of 5	196	29
	≥4 out of 5	479	71

**Table 2:** The descriptive analysis of the medical students’ sleep, dietary, and exercise habits. *N* = 675.

Variables		Frequency	Proportion
Daily sleep hours	<4 h	24	3.6
	4–6 h	311	46.1
	6–8 h	280	41.5
	>8 h	60	8.9
Sleep satisfaction the previous month	Very dissatisfied	75	11.1
	Dissatisfied	226	33.5
	Neither	205	30.4
	Satisfied	124	18.4
	Very satisfied	45	6.7
Falling asleep easily over the previous month	Very difficult	27	4
	Difficult	92	13.6
	Neutral	193	28.6
	Easy	232	34.4
	Very easy	131	19.4
Daily meals	Breakfast and dinner	82	12.1
	Breakfast and lunch	124	18.4
	Breakfast only	7	1
	Breakfast, lunch, and dinner	190	28.1
	Dinner only	28	4.1
	Lunch and dinner	190	28.1
	Lunch only	54	8
Fast food consumption	Never	8	1.2
	Rarely	107	15.9
	Sometimes	260	38.5
	Usually	216	32
	Always	84	12.4
Daily drinking of 2 L of water	Never	23	3.4
	Rarely	153	22.7
	Sometimes	197	29.2
	Usually	169	25
	Always	133	19.7
Skipping breakfast	Never	93	13.8
	Rarely	158	23.4
	Sometimes	155	23
	Usually	152	22.5
	Always	117	17.3
Drinking coffee per day	Never	58	8.6
	Rarely	82	12.1
	Sometimes	108	16
	Usually	108	16
	Always	319	47.3
Exercising ≥30 min	Never	113	16.7
	Very rarely	168	24.9
	Once per Month	38	5.6
	Few times per month	81	12
	About once a week	58	8.6
	A few times per week	131	19.4
	Five days a week	57	8.4
	Daily	29	4.3

**Table 3:** The descriptive analysis of the medical students’ social activity and smoking habits. *N* = 675.

Variables		Frequency	Proportion
Social status	Introvert	439	65
	Extrovert	236	35
	Not satisfied	127	18.8
	Somewhat satisfied	295	43.7
	Satisfied	253	37.5
Cigarette smoking	Never	586	86.8
	Rarely	40	5.9
	Once per week	7	1
	Few times per week	11	1.6
	Daily	31	4.6
Electronic cigarette	Never	582	86.2
	Rarely	26	3.9
	Once per week	4	0.6
	Few times per week	15	2.2
	Daily	48	7.1
Shisha smoking	Never	583	86.4
	Rarely	77	11.4
	Once per week	11	1.6
	Few times per week	2	0.3
	Daily	2	0.3

**Table 4:** Bivariate analysis of lifestyle characteristics across sex. *N* = 675.

Variables		Student Sex Frequency (Proportion)			
		Female	Male	Test Statistic	*p*-Value
Level of study	Pre-clinical phase (years 1–3)	145 (48.2)	126 (33.7)	χ^2^(1) = 14.64	<0.001
	Clinical phase (4th–6th years)	156 (51.8)	248 (66.3)		
Meals daily	Breakfast and dinner	36 (12)	46 (12.3)	χ^2^ (6) = 51.64	<0.001
	Breakfast and lunch	83 (27.6)	41 (11)		
	Breakfast only	6 (2)	1 (0.3)		
	Breakfast, lunch, and dinner	71 (23.6)	119 (31.8)		
	Dinner only	14 (4.7)	14 (3.7)		
	Lunch and dinner	60 (19.9)	130 (34.8)		
	Lunch only	31 (10.3)	23 (6.1)		
Fast food consumption	Never	6 (2)	2 (0.5)	χ^2^ (4) = 23.4 LR	<0.001
	Rarely	57 (18.9)	50 (13.4)		
	Sometimes	125 (41.5)	135 (26.1)		
	Usually	93 (30.9)	123 (32.9)		
	Always	20 (6.6)	64 (17.1)		
Daily drinking 2 L of water	Never	17 (5.6)	6 (1.6)	χ^2^ (4) = 45.61	<0.001
	Rarely	93 (30.9)	60 (16)		
	Sometimes	95 (31.6)	102 (27.3)		
	Usually	56 (18.6)	113 (30.2)		
	Always	40 (13.3)	93 (24.9)		
Coffee consumption per day	Never	20 (6.6)	38 (10.2)	χ^2^ (4) = 21.45	<0.001
	Rarely	31 (10.3)	51 (13.6)		
	Sometimes	39 (13.0)	69 (18.4)		
	Usually	39 (13.0)	69 (18.4)		
	Always	172 (57.1)	147 (39.3)		
Satisfied/Happy with social life	Not satisfied	71 (23.6)	56 (15)	χ^2^ (2) = 14.08	0.001
	Somewhat satisfied	138 (45.8)	157 (42.0)		
	Satisfied	92 (30.6)	161 (43.0)		
Cigarette smoking	Never	276 (91.7)	310 (82.9)	χ^2^ (4) = 17.58 LR	0.001
	Rarely	16 (5.3)	24 (6.4)		
	Once per week	1 (0.3)	6 (1.6)		
	Few times per week	3 (1.0)	8 (2.1)		
	Daily	5 (1.7)	26 (7.0)		
Electronic cigarette smoking	Never	276 (91.7)	306 (81.8)	χ^2^ (4) = 26.75 LR	<0.001
	Rarely	13 (4.3)	13 (3.5)		
	Once per week	1 (0.3)	3 (0.8)		
	Few times per week	5 (1.7)	10 (2.7)		
	Daily	6 (2.0)	42 (11.2)		
